# Testing the Accuracy of Eukaryotic Phylogenetic Profiles for Prediction of Biological Function

**DOI:** 10.4137/ebo.s863

**Published:** 2008-06-18

**Authors:** Saurav Singh, Dennis P. Wall

**Affiliations:** Center for Biomedical Informatics, Harvard Medical School, Boston, MA 02115

**Keywords:** comparative genomics, phylogenetic profiles, functional prediction

## Abstract

A phylogenetic profile captures the pattern of gene gain and loss throughout evolutionary time. Proteins that interact directly or indirectly within the cell to perform a biological function will often co-evolve, and this co-evolution should be well reflected within their phylogenetic profiles. Thus similar phylogenetic profiles are commonly used for grouping proteins into functional groups. However, it remains unclear how the size and content of the phylogenetic profile impacts the ability to predict function, particularly in Eukaryotes. Here we developed a straightforward approach to address this question by constructing a complete set of phylogenetic profiles for 31 fully sequenced Eukaryotes. Using Gene Ontology as our gold standard, we compared the accuracy of functional predictions made by a comprehensive array of permutations on the complete set of genomes. Our permutations showed that phylogenetic profiles containing between 25 and 31 Eukaryotic genomes performed equally well and significantly better than all other permuted genome sets, with one exception: we uncovered a core of group of 18 genomes that achieved statistically identical accuracy. This core group contained genomes from each branch of the eukaryotic phylogeny, but also contained several groups of closely related organisms, suggesting that a balance between phylogenetic breadth and depth may improve our ability to use Eukaryotic specific phylogenetic profiles for functional annotations.

## Introduction

A phylogenetic profile is a binary representation of a gene’s evolution through time. When the evolutionary pattern of a gene’s gains and losses closely matches that of another, it is plausible to assume that these two genes have coevolved and coordinate to perform a biological function. Phylogenetic profiles have become critical for various pursuits in comparative genomics and systems biology including functional prediction (Pellegrini et al. 1999; Wu et al. 2003; Gaasterland and Ragan, 1998), cellular localization of proteins (Marcotte et al. 2000), and the construction of regulatory networks within the cell (Bowers et al. 2004; Wu et al. 2006; Date and Marcotte, 2003). By and large, the successes have come from the use of phylogenetic profiles composed entirely of bacterial genomes, and thus the extensibility of the approaches and conclusions to Eukaryotes remains unclear. Many of these research studies did not address the question of how genome composition and number impact the utility of phylogenetic profiles. However, with the dramatic rise of fully sequenced genomes, in particular Eukaryotic genomes, these questions have taken center stage, chiefly because of the importance of bioinformatics approaches like phylogenetic profiling for rapid and accurate genome annotation and network construction.

As a consequence, new research has emerged that addresses how genome content and number alter the predictive power of phylogenetic profiles both with and without a sizeable collection of Eukaryotes (Jothi et al. 2007; Snitkin et al. 2006; Sun et al. 2007; Sun et al. 2005). Two of these important studies (Jothi et al. 2007; Snitkin et al. 2006) assembled large groups of both bacterial and Eukaryotic genomes to assess performance in general, and the effect of Eukaryotes on performance in particular. Both concluded that Eukaryotic genomes significantly degrade performance and call into question the application of phylogenetic profiles for functional annotation of Eukaryotic genomes.

In the present study, we expand upon this previous research by focusing exclusively on Eukaryotes, in particular by testing numerous combinations of Eukaryotic genomes to find reference sets that exhibited optimal accuracy of functional prediction. To this end, we conducted a comprehensive set of permutations on a set of 31 Eukaryotic genomes by knocking out, one-by-one, conspicuous outlier genomes. This strategy generated 30 different genome sets of decreasing size and variable genome composition and provided a global view of accuracy and coverage of functional predictions. Our results show that Eukaryotic phylogenetic profiles can be used for the study of function in Eukaryotic genomes, but that accuracy and coverage are both highly dependent on the specific genomes used.

## Methods

### Assembling phylogenetic profiles

We started with a complete set of phylogenetic profiles built from ortholog analysis among the 31 Eukaryotic genomes currently available in the web-enabled tool RoundUp (DeLuca et al. 2006; Wall et al. 2003). Using gene ontology (GO) (Harris et al. 2004), we assigned biological processes to every profile and grouped profiles annotated with the same process. We allowed single profiles to be represented in multiple processes. In an attempt to account for the variability of gene annotation and to minimize redundancy of GO terms among the processes, we grouped GO identifiers together if they were found along the same direct path to the root of the biological process ontology. Terms close to the root, specifically at levels 1 and 2, were removed. At the time of writing, this procedure amounted in 886 distinct GO process subgraphs, hereafter referred to as functional modules.

### Comparing and permuting phylogenetic profiles

To assess the ability of a particular permutation (number and type of genomes within a phylogenetic profile) to predict function, we first computed a hamming distance for all pairs of phylogenetic profiles in every functional module to form k_in_. We then computed the pairwise hamming distance matrix for all members of a specific functional module against all members of another functional module to form k_out_ for all functional modules. A student’s t-test was used to determine whether k_in_ and k_out_ differed significantly. Since each functional module’s k_in_ must be compared to the 885 other functional modules, the p values from the t-test were adjusted by the false discovery rate to generate q values (Storey 2002). The q value is a measurement of significance framed in terms of the false discovery rate, rather than the rate of false positives. It has the benefit of being less conservative than alternative approaches to multiple test correction while still minimizing the number of false positive findings. Values below 0.05 were taken as sufficient evidence that the two functional modules being compared could be distinguished by their phylogenetic profiles.

Beginning with all 31 Eukaryotic genomes, we successively removed one at a time—the outlier genome causing the largest increase in k_in_—until only 2 genomes remained, amounting in 30 different phylogenetic profile permutations. After each deletion we computed and compared k_in_ and k_out_ to generate a complete set of q values for all phylogenetic profile permutations.

To test whether changes in the predictive power of a permutation were due to alterations in size or genome composition we used the Kolmogorov-Smirnov (KS) test. The KS test is a nonparametric test that assesses the difference between two distributions, assuming as the null hypothesis that the two samples are distributed identically. In our case, the samples compared were the numbers of significant q values obtained by comparing k_in_ and k_out_ for all 886 functional modules. A KS test was performed for all-against-all phylogenetic profile permutations; a p value <0.05 was considered evidence that the accuracy of the two genome sets was significantly different.

## Results

**Figure 1:**
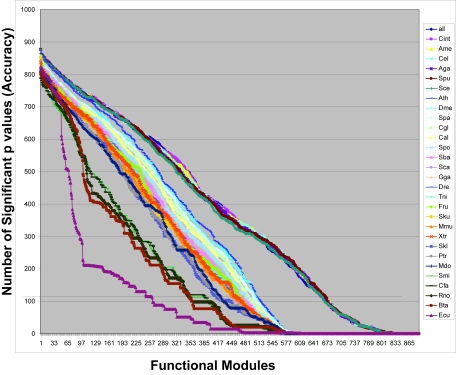
The functional prediction accuracy of phylogenetic profiles from 30 different eukaryotic genome sets. Accuracy was determined by t-test comparison of average hamming distance for phylogenetic profiles within a functional module versus phylogenetic profiles in all 885 other functional modules. P values were adjusted to account for multiple testing by the method described in (Storey, 2002). The plot depicts the number of corrected p values per functional module and demonstrates that there are 4 zones of decreasing accuracy within which the level of performance of each phylogenetic profile for predicting function is statistically indistinguishable, but between which the performance declines significantly.

We constructed 21,706 Eukaryotic phylogenetic profiles using orthologs calculated by RoundUp (DeLuca et al. 2006) and employed an algorithm to remove outlier genomes one at a time until two genomes remained, amounting in 30 different permutations. In all permutations, the outlier was obvious and no ties were encountered. For each permutation the difference between k_in_ and k_out_ for all 886 functional modules was tested. The number of significant FDR-adjusted q values (q < 0.05) per phylogenetic profile permutation was considered a direct measurement of the accuracy of each genome set for predicting membership in a gene ontology-defined functional module. The results of this analysis for the 30 different permutations are displayed in Figure 1 (raw data available online as Supplementary Table 1), and Table 1 lists the order and identity of genome deletions per permutation. The greatest accuracy was near 99%, but this was true for only a single functional module (GO:0000751, “cell cycle arrest in response to pheromone”).

In general, bigger genome sets were better at predicting membership in a functional module than smaller ones (Fig. 1). However, most of the permutations predicted at least a fraction of the functional modules. In all but three (permutations Ecu, Cfa, Smi corresponding to genome set sizes of 2, 5, and 6, respectively), 50 or more specific functional modules could be predicted with 80% or higher accuracy. Several of these well predicted functional modules were identified by more than one permuted genome set, indicating that the predictive power of genome sets overlapped in certain cases. Specifically, a total of 14 functional modules were accurately distinguished by all permuted genome sets (Table 2) and 27 functional modules were found in the top 50 in 20 or more of the 30 permutations. Each permutated genome set was also able to uniquely identify certain functional modules. By manual inspection, these appeared to be derived pathways that have evolved in a subset of the eukaryotic organisms. The union of all functional modules predicted at 80% or higher accuracy contained 203 distinct members (available online as Supplementary Table 2). Several of the functional modules predicted with 80% or better accuracy contained over 200 phylogenetic profiles, significantly larger than the mean size of 12.8 profiles per module. This indicates that accuracy levels were not artificially inflated by modules with few genes and/or genomes.

**Figure 2:**
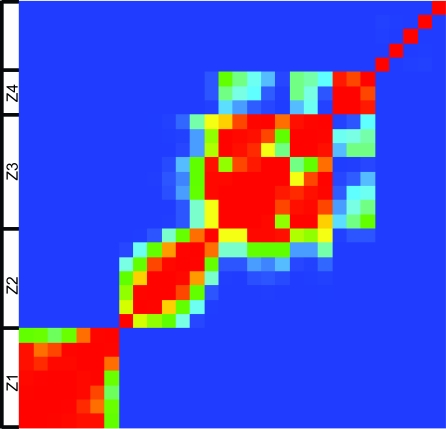
Heatmap showing significant differences in the accuracy of the functional predictions made by 30 different genome sets tested in the present study. Significance was measured by Kolmogorov-Smirnov (KS) tests. Red indicates an insignificant p value = 1, Blue represents a significant p value = 0. The 4 large zones of accuracy also evident in Figure 1 are shown here to differ significantly with all KS-test p values < 0.01 (Z1–Z4).

The predictive power did not decline linearly with the successive deletion of genomes. Instead, significant decreases in accuracy were seen only after deleting certain genomes, the most obvious changes occurring with the 7th (Ath), the 13th (Sba), and the 22nd (Xtr). These single genome alterations to the genome sets formed visible zones of accuracy when graphed (Fig. 1). By KS tests we determined that the different genome sets within a zone had statistically indistinguishable accuracy for predicting membership in a functional module, but that each zone was significantly different from the others (Fig. 2; raw data available online in Supplementary Table 3). This demonstrated that within zones neither the genome content nor the size of the genome set altered the utility of the phylogenetic profile for predicting function.

We then questioned whether the differences in accuracy across the zones were due to a specific genome being added or deleted. For example, a reasonable hypothesis was that the addition or deletion of Arabidopsis thaliana (Ath) caused the sharp change in accuracy between profiles containing 25 versus those containing 24 genomes. To address this question, we ran permutations to delete any and all combinations of the first seven largest outlier genomes including Ath, and compared the accuracy of each permuted genome set against the accuracy conferred by the complete set of 31 genomes using KS tests. This analysis demonstrated that as many as six could be deleted randomly without altering the accuracy, but that at least one of the seven must be included in the genome set to retain the accuracy equal to the complete genome set.

To test whether our strategy for altering the genome set by first deleting the largest outlier genomes introduced bias into our estimates of predictive power, we successively deleted the smallest genomes one-by-one and compared their predictive power against that found using the complete set of genomes. Similar to the scenario observed when deleting the largest outliers, only after the 8th smallest outlier genome was deleted did a significant decline in performance occur. This suggested the possibility that the genome in particular, in this case Smi, contributed more to the accuracy of the phylogenetic profile than the other 7. However this turned out to be false. By running additional permutations to test any and all combinations of the 8 smallest outlier genomes plus the remainder, we discovered that as many as 7, chosen at random, could be deleted without altering the accuracy of the genome set for functional prediction.

The findings that all but one of the 7 largest outlier genomes and all but one of the 8 smallest outlier genomes could be deleted without negatively impacting the predictive power of the phylogenetic profiles provoked us to test if removal of all 13 genomes to generate a set of 18 would yield equal predictive power to the genome set containing all 31. Indeed, deleting all 13 achieved results statistically indistinguishable from those found by the genome set with no deletions, regardless of which one of the largest and smallest outliers were retained in the genome set (average p value for these tests was 0.8). Thus, the following genome set equaled the predictive performance of the largest, and most accurate, genome sets tested:

### X, Dme, Spa, Cgl, Cal, Spo, Sba, Sca, Gga, Dre, Tni, Fru, Sku, Mmu, Xtr, Skl, Ptr, Y

Where X and Y are any of the seven largest (Cint, Ame, Cel, Aga, Spu, Sce, Ath) and eight smallest (Mdo, Smi, Cfa, Rno, Bta, Ecu, Hsa, Mus) outlier genomes, respectively. Any additional genome deletions from this core set of 18 caused a significant decline in the accuracy.

## Discussion

Previous research has shown that size and composition of phylogenetic profiles dramatically alter their ability to predict function and that the Eukaryotic genomes have a significant negative impact on performance, most likely because of the bacterial contributions that occurred during the evolution of Eukaryotes as well as the over-representation of both parasitic unicellular Eukaryotes and vertebrates among the set of fully sequenced genomes (Jothi et al. 2007). In the present study, we probed this issue more deeply by building phylogenetic profiles composed only of Eukaryotes. We tested a large variety of genome sets using gene ontology as our gold standard to determine how well profiles could predict function in general and, in particular, how the size and composition altered the predictive power.

We found that the profiles composed of larger numbers of genomes, between 25 and 31, had the greatest power, predicting on average 30% of the functional modules with 70% accuracy. A significant decline in accuracy was found when the seven largest outliers were deleted from the complete set of 31 genomes. The cause of this degradation in performance appeared to be mainly due to the reduction of the genome set from 25 to 24 genomes, than to any specific genome being deleted, as the addition of any one of the seven restored the accuracy to the level exhibited by the largest profiles. This further suggested that the seven largest outliers (Cint, Ame, Cel, Aga, Spu, Sce, Ath) contain redundant information for functional prediction, a finding that was difficult to explain as they are widely dispersed across the phylogeny of Eukaryotes.

We also discovered that the 8 smallest outliers are redundant with respect to their ability to contribute to the prediction of membership in a functional module. While the deletion of all 8 significantly degraded the accuracy of functional predictions, insertion of any one to the genome set containing the remaining 23 genomes restored the accuracy to the highest levels found. Unlike the situation with the 7 largest outliers, the phylogenetic distribution of the 8 of the smallest outliers (Mdo, Smi, Cfa, Rno, Bta, Ecu, Hsa, Mus) may explain the reason for the large degree of overlap in predictive power, given that a majority of them are mammals.

Together these two results suggested that all but one of the largest and one of the smallest outliers could be removed from the complete set of 31 to generate a small core that has equal ability to predict function as the best performing genomes sets. This hypothesis was supported by our results—a total of 13 genomes could be deleted without altering the predictive power. Furthermore, any additional deletions from this “Eukaryotic core” significantly degraded performance. This core group contained genomes from each branch of the Eukaryotic phylogeny, but also contained several groups of closely related organisms, suggesting that a balance between phylogenetic breadth and depth may improve our ability to use Eukaryotic specific phylogenetic profiles for functional annotations.

The results from all of our permutations indicate that the size of the phylogenetic profile is important for high precision and recall of functional prediction, an expected outcome given that larger profiles have more possible patterns. However, the size of the profile only matters to a point, beyond which the addition of new genomes yields no additional resolution (e.g. adding more of the largest or smallest outliers did not significantly increase accuracy or coverage). In the present study, we determined that it was not only important to have 18 genomes in the reference set, but that the choice of genomes was critical for achieving maximum performance. Similarly, Jothi et al. (2007) concluded that not only is the number of genomes important, but that careful selection of informative genomes in the reference set impacts the accuracy of phylogenetic profiles.

Although the Eukaryotic core may represent a best-case solution to achieve both optimal accuracy and coverage using a single reference set, nearly all of the permuted genome sets could predict 50 or more functional modules with 80% or higher accuracy (clustering evident in the upper left of Fig. 1). Furthermore, many of these well predicted functional modules were uniquely identified by only one or a few of the genome sets, suggesting that even higher coverage without loss of accuracy could be achieved by combining predictions from numerous reference sets, however a more precise understanding of the classes of functions that are best predicted by different reference sets would be required before such groupings could be done effectively.

In the present analysis, we elected to use the reciprocal smallest distance algorithm (Wall et al. 2003) to detect orthologs, because it uses global sequence alignment and maximum likelihood estimation of evolutionary distances to detect orthologs and is thus less likely to be misled by the presence of close paralogs than approaches that rely on reciprocal best blast hits (e.g. (Sun et al. 2005)). However, there is no question that the genomic complexity of Eukaryotes, including extensive amounts of domain shuffling and gene family evolution, can complicate the search for truly functionally equivalent genes and therefore negatively impact the utility of phylogenetic profiles for predicting protein function (Ranea et al. 2007). Future work to assess prediction performance of profiles that have been constructed by alternative methods should greatly improve our understanding of how the shape and size of phylogenetic profiles impact our ability to predict protein function in Eukaryotes. For example, domain based approaches (Ranea et al. 2007) should increase the ability to predict membership in functional modules that would otherwise be missed by approaches like to one presented here.

## Figures and Tables

**Table 1:** Order and identity of genomes deletions for the 30 permutations depicted in Figure 1. Genomes are in decreasing order from largest to smallest outlier. Phylogenetic profiles for a given genome set were grouped into biological processes defined by gene ontology; a pairwise distance matrix was then generated to identify the outlier genome (no ties were discovered). To evaluate the affect of size and composition on the power of a phylogenetic profile to predict function, outlier genomes were removed one-by-one until the size of the genome set equaled 2, i.e. composed of just Hsa and Mus.

*Ciona intestinalis*	Cint
*Apis mellifera*	Ame
*Caenorhabditis elegans*	Cel
*Anopheles gambiae*	Aga
*Strongylocentrotus purpuratus*	Spu
*Saccharomyces cerevisiae*	Sce
*Arabidopsis thaliana*	Ath
*Drosophila melanogaster*	Dme
*Saccharomyces paradoxus*	Spa
*Candida glabrata*	Cgl
*Candida albicans*	Cal
*Schizosaccharomyces pombe*	Spo
*Saccharomyces bayanus*	Sba
*Saccharomyces castellii*	Sca
*Gallus gallus*	Gga
*Danio rerio*	Dre
*Tetraodon nigroviridis*	Tni
*Fugu rubripes*	Fru
*Saccharomyces kudriavzevii*	Sku
*Macaca mulatta*	Mmu
*Xenopus tropicalis*	Xtr
*Saccharomyces kluyveri*	Skl
*Pan troglodytes*	Ptr
*Monodelphis domestica*	Mdo
*Saccharomyces mikatae*	Smi
*Canis familiaris*	Cfa
*Rattus norvegicus*	Rno
*Bos taurus*	Bta
*Encephalitozoon cuniculi*	Ecu
*Homo sapiens*	Hsa*
*Mus Musculus*	Mus*

**Table 2:** Functional modules predicted with 80% or higher accuracy by the 30 genome sets tested in the present study. A functional module includes all Gene Ontology (GO) terms along the path to the root of the GO process ontology excluding those closest to the root, specifically terms at levels 1 and 2.

Parent GO ID	Biological Process Description	Size of Module	# Profiles in Subgraph
GO:0048691	positive regulation of axon extension involved in regeneration	24	309
GO:0048478	replication fork protection	32	267
GO:0048128	oocyte axis determination, oocyte nuclear migration	43	274
GO:0046638	positive regulation of alpha-beta T cell differentiation	69	687
GO:0045500	sevenless signaling pathway	30	378
GO:0045082	positive regulation of interleukin-10 biosynthetic process	73	677
GO:0043306	positive regulation of mast cell degranulation	15	223
GO:0042776	mitochondrial ATP synthesis coupled proton transport	29	291
GO:0035067	negative regulation of histone acetylation	44	660
GO:0035056	negative regulation of nuclear mRNA splicing via U2-type spliceosome	22	216
GO:0030702	chromatin silencing at centromere	33	371
GO:0008377	light-induced release of internally sequestered calcium ion	47	482
GO:0007253	cytoplasmic sequestering of NF-kappaB	78	569
GO:0000752	agglutination during conjugation with cellular fusion	10	203
